# Phosphoinositide Signaling and Actin Polymerization Are Critical for Tip Growth in the Marine Red Alga *Pyropia yezoensis*

**DOI:** 10.3390/plants14142194

**Published:** 2025-07-15

**Authors:** Ryunosuke Irie, Koji Mikami

**Affiliations:** 1 Fisheries Promotion Division, Agriculture, Forestry and Fisheries Department, Oita Prefectural Government, 3-1-1 Otemachi, Oita 870-8501, Japan; irie-ryunosuke@pref.oita.lg.jp; 2 Department of Integrative Studies of Plant and Animal Production, School of Food Industrial Sciences, Miyagi University, 2-2-1 Hatatate, Taihaku-ku, Sendai 982-0215, Japan

**Keywords:** tip growth, branch formation, stem cell, actin polymerization, phosphoinositide-phosphate 3-kinase, phospholipase C, conchocelis, *Pyropia yezoensis*

## Abstract

In the marine red alga *Pyropia yezoensis*, filamentous phases of the life cycle, e.g., the conchocelis (sporophyte) and conchosporangium (conchosporophyte), proliferate by tip growth. In this study, we investigated the possible involvement of phosphoinositide turnover and actin polymerization in the spontaneous initiation and tip growth of new branches in isolated single-celled conchocelis cells using pharmacological treatments. Treatment with LY294002 and U73122, specific inhibitors of phosphoinositide-phosphate 3-kinase and phospholipase C, respectively, reduced side-branch formation and inhibited the elongation of branches. In addition, two inhibitors of the actin cytoskeleton, cytochalasin B (CCB) and latrunculin B (LAT-B), had similar effects on tip growth. However, CCB did not alter the branching rate of single-celled conchocelis, whereas LAT-B did. As CCB and LAT-B affect actin polymerization through different mechanisms, this result suggests differences in the contributions of actin polymerization to branch initiation versus tip growth. These findings demonstrate the critical and diverse functional roles played by phosphoinositide turnover and actin polymerization in the regulation of the initiation and maintenance of tip growth in the conchocelis phase of *P. yezoensis*.

## 1. Introduction

Tip growth is a type of cellular growth occurring in filamentous bodies and tissues such as fungal hyphae, angiosperm pollen tubes and root hairs, moss protonemata, green algal rhizoids, and thali of filamentous brown algae [1,2,3]. The mechanisms regulating tip growth have been analyzed extensively in the tip-growing cells of angiosperms, namely, root hairs and pollen tubes that are non-dividing single cells with elongation occurring at the tip, and they involve ion fluxes [4,5], asymmetric distributions of F-actin [4,5,6,7] and phosphoinositides [4,8,9,10], trafficking of membrane and cell wall materials [4,5,11], and production of plant hormones [4,12,13]. Similar observations have been made for tip growth in hyphae and protonemata of filamentous multicellular organisms that grow via cell division restricted at the tip stem cells [1,2,6]. In single-cell and hyphae types of tip growth, modulations of ion fluxes, actin polymerization, phosphoinositide turnover, and membrane trafficking commonly occur at the apex of the growing cells. This establishes a restricted point of growth that polarizes growth direction and leads to tip growth, for which phosphoinositide turnover promotes Ca^2+^ flux to activate Ca^2+^-dependent physiological regulations, accelerates membrane trafficking of membrane and cell wall components for exsocytosis, and establishes asymmetrical distribution of some phosphatidylinositols on plasma membrane. Further, certain phosphatidylinositols bind actin-binding proteins to reorganize actin distribution at the apex of tip cells [5,7,14,15].

The marine red alga *Pyropia yezoensis* (Bangiales, Rhodophyta) is an important species for the nori industry in Asian countries such as Japan, South Korea, and China [16]. Despite being cultivated in industrial large-scale aquaculture systems on the sea surface, *P. yezoensis* is relatively poorly studied; thus, little is known about the mechanisms regulating its growth, life cycle, development, and environmental stress responses. However, we have recently determined that tip growth is used for proliferation during filamentous phases of the *P. yezoensis* life cycle, e.g., the conchocelis (sporophyte) and conchosporangium (conchosporophyte) [17,18]. In these phases, elongation and division occur only in the apical cell of the filament, and each cell division produces two different cell types: a new apical cell at the tip of the filament and a neighboring differentiated non-dividing cell [17,18]. Similar hyphae-type tip growth was also observed in the gametophytic protonema of the moss *Physcomitrium patens*, where the tip cell is now recognized as a stem cell that divides to produce a copy of itself and a differentiated non-dividing cell [19]. Thus, tip cells of the conchocelis and conchosporangium of *P. yezoensis* also are considered to be stem cells. Therefore, elucidation of the mechanisms regulating the production and maintenance of the apical stem cell is fundamental for understanding tip growth as a growth mode of the two filamentous phases in the *P. yezoensis* life cycle.

In addition to polarized tip growth in the conchocelis and conchosporangia, directional migration of asexual spores from leafy gametophytes is polarity-dependent in *P. yezoensis* [20,21]. Establishment of polarity along the anterior–posterior axis is regulated by phosphoinositide-phosphate 3-kinase (PI3K) and phospholipase C (PLC). PI3K phosphorylates a hydroxyl group (-OH group) at the third position of the inositol ring of phosphoinositide (PtdIns), phosphoinositide 4-phosphate, phosphoinositide 5-phosphate, and phosphoinositide 4,5-bisphosphate [PtdIns(4,5)P2] to generate phosphoinositide 3-phosphate, phosphoinositide 3,4-bisphosphate, phosphoinositide 3,5-bisphosphate, and phosphoinositide 3,4,5-trisphosphate, respectively. PLC hydrolyzes PtdIns(4,5)P2 to produce two second messengers, inositol 1,4,5-trisphosphate and diacylglycerol [22,23]. Phosphoinositide turnover mediated by these enzymes is critical for the directional migration of asexual spores of *P. yezoensis*. In addition, F-actin is distributed asymmetrically to the forward side of migrating cells [20,21]. These findings led us to hypothesize that PI3K, PLC, and F-actin are involved in polarized tip growth in *P. yezoensis*.

We previously developed a novel experimental system using single-celled conchocelis for the study of tip growth in *P. yezoensis* [18]. This system enabled us to identify the factors involved in the generation of a branch initial from differentiated non-dividing cells, tip growth of the branch initial, and establishment and maintenance of the stem cell by cell elongation and cell division. In this study, we addressed whether phosphoinositide turnover and actin polymerization are critical for tip growth in single-celled conchocelis from *P. yezoensis*. Our findings provide novel insights into the regulation of hyphae-type tip growth.

## 2. Results

### 2.1. Effects of PI3K and PLC Inhibitors

**Figure 1:**
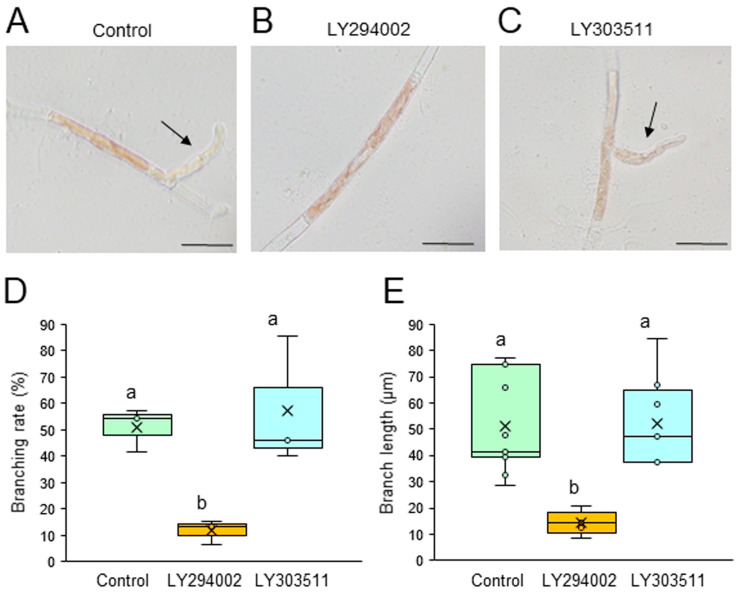
Effects of a PI3K inhibitor on tip growth. (**A**–**C**) Photographs of single conchocelis cells treated with 0.5% DMSO as a control (**A**), 10 μM LY294002 (**B**), or its inactive analog 10 μM LY303511 (**C**) for 3 days. Arrows indicate newly generated branches. Bars: 25 μm. (**D**,**E**) Branching rate (**D**) and branch length (**E**) following the treatment of single conchocelis cells with 0.5% DMSO (control), 10 μM LY294002, or 10 μM LY303511 for 3 days. Center line, median line; box limits, interquartile range with upper and lower quartiles; points, data; whiskers, range with maximum and minimum values; crosses, mean value. Lowercase letters denote significant differences in branching rate (**D**) and branch length (**E**) based on three independent experiments (*n* = 3), as determined by the Tukey−Kramer test (*p* < 0.05) for each set of treatments.

**Figure 2:**
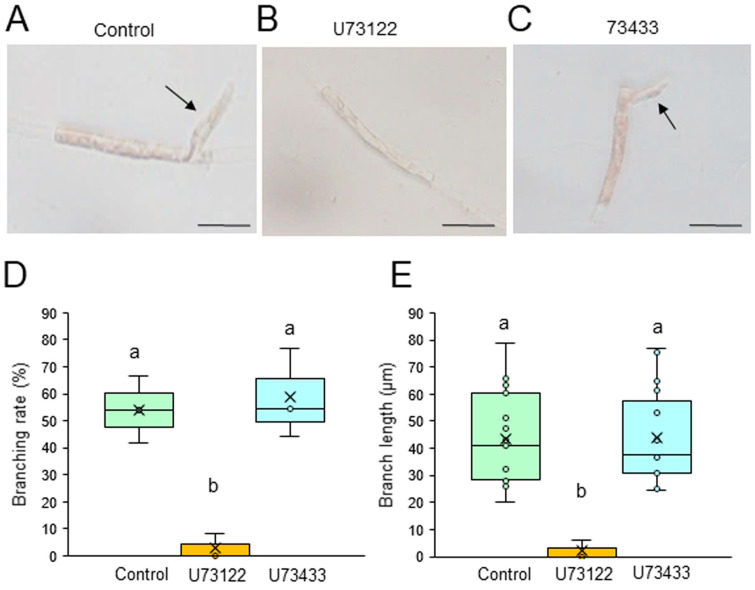
Effects of a PLC inhibitor on tip growth. (**A**–**C**) Photographs of single conchocelis cells treated with 0.5% DMSO as a control (**A**), 0.1 μM U73122 (**B**), or its inactive analog 0.1 μM U73433 (**C**) for 3 days. Arrows indicate newly generated branches. Bars: 25 μm. (**D**,**E**) Branching rate (**D**) and branch length (**E**) following treatment of single conchocelis cells with 0.5% DMSO (control), 0.1 μM U73122, or 0.1 μM U73433 for 3 days. Center line, median line; box limits, interquartile range with upper and lower quartiles; points, data; whiskers, range with maximum and minimum values; crosses, mean value. Lowercase letters denote significant differences in branching rate (**D**) and branch length (**E**) based on three independent experiments (*n* = 3), as determined by the Tukey−Kramer test (*p* < 0.05) for each set of treatments.

We first examined the possible involvement of PI3K and PLC in the generation of branch initials and their subsequent tip growth using chemical inhibitors of these enzymes. Single-celled conchocelis cells were treated with 0, 5, or 10 μM LY294002, a specific inhibitor of PI3K, or LY303511, its inactive analog, for 3 days. The branching rate and branch length were lower in the presence of LY294002 in a dose-dependent manner (Figure S1), with strong inhibition at 5 μM; however, LY303511 had no significant effect, even at 10 μM (Figure 1). Similarly, a 3-day treatment of single-celled conchocelis with U73122, a specific inhibitor of PLC, inhibited both the branching rate and branch length in a dose-dependent manner (Figure S2), whereas these effects were not observed after using U73433, an inactive analog of U73122 (Figure 2). Thus, we concluded that PI3K and PLC activities are important for the generation and tip growth of branch initials from differentiated conchocelis cells.

### 2.2. Effects of Actin Polymerization Inhibitors

**Figure 3:**
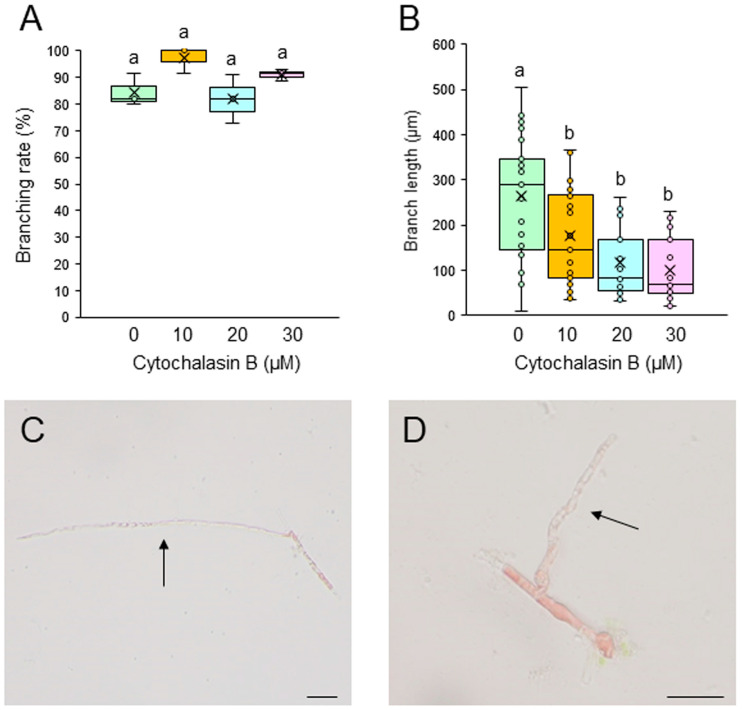
Effects of the actin polymerization inhibitor cytochalasin B (CCB) on tip growth. (**A**,**B**) Branching rate (**A**) and branch length (**B**) following the treatment of single conchocelis cells with 0.5% DMSO (control) or 10, 20, or 30 μM CCB for 7 days. Center line, median line; box limits, interquartile range with upper and lower quartiles; points, data; whiskers, range with maximum and minimum values; crosses, mean value. Lowercase letters denote significant differences in branching rate (**A**) and branch length (**B**) based on three independent experiments (*n* = 3) as determined by the Tukey-Kramer test (*p* < 0.05) for each set of treatments. (**C**,**D**) Photographs of single-celled conchocelis treated with 0.5% DMSO (**C**) or 20 μM CCB (**D**) for 7 days. Arrows indicate newly generated branches. Bars: 50 μm.

**Figure 4:**
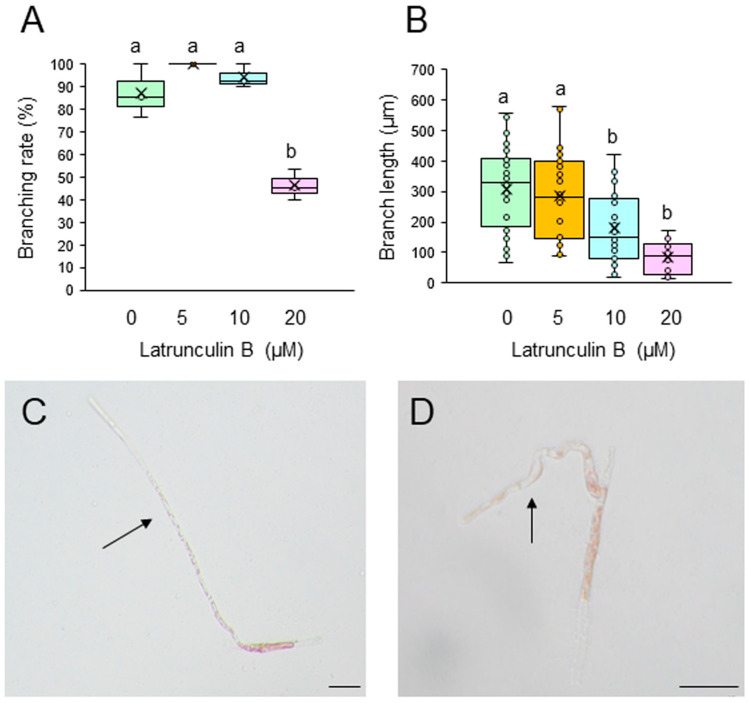
Effects of the actin polymerization inhibitor latrunculin B (LAT-B) on tip growth. (**A**,**B**) Branching rate (**A**) and branch length (**B**) following treatment of single-celled conchocelis with 0.5% DMSO (control) or 5, 10, or 20 μM LAT-B for 7 days. Center line, median line; box limits, interquartile range with upper and lower quartiles; points, data; whiskers, range with maximum and minimum values; crosses, mean value. Lowercase letters denote significant differences in branching rate (**A**) and branch length (**B**) based on three independent experiments (*n* = 3) as determined by the Tukey−Kramer test (*p* < 0.05) for each set of treatments. (**C**,**D**) Photographs of single-celled conchocelis treated with 0.5% DMSO (**C**) or 20 μM LAT-B (**D**) for 7 days. Arrows indicate newly generated branches. Bars: 50 μm.

To test whether F-actin is involved in the generation and tip growth of branch initials, single-celled conchocelis was treated with different concentrations of the actin cytoskeleton inhibitors cytochalasin B (CCB) and latrunculin B (LAT-B). Incubation of single conchocelis cells with 10, 20, or 30 μM CCB for 7 days did not affect the branching rate (Figure 3A). In contrast, the same treatments resulted in a shorter branch length (Figure 3B), and the branches displayed a wavy shape (compare Figure 3C,D). When the cells were incubated with 5, 10, or 20 μM LAT-B for 3 or 7 days, the branching rate and branch length were inhibited in a dose-dependent manner (Figure 4A,B) and, as observed with the CCB treatments, the branches were wavy (compare Figure 4C,D). When LAT-B was removed by washing the cells with seawater, we observed recovery of both the branching rate and branch growth (Figure S3). Therefore, although the effects of the two inhibitors differed, our experimental results indicate the involvement of F-actin in the generation of branch initials, branch tip growth, and the establishment of stem cells in conchocelis cells.

## 3. Discussion

This study demonstrated that PI3K and PLC activities and actin polymerization have critical roles in the generation and tip growth of branch initials in single-celled conchocelis of *P. yezoensis*. These findings are consistent with the mechanisms of tip growth in the pollen tubes and root hairs of terrestrial plants, where more detailed analyses have been performed, including pharmacological inhibition of PI3K and PLC [24,25], overexpression and knockdown of genes encoding PLCs and PIP5Ks [24,26,27,28,29,30,31], and visualization of the subcellular location of phosphoinositide, PLC, and F-actin [24,32,33,34,35,36]. *P. yezoensis* lacks a reverse-genetic experimental system that could provide direct evidence for the functions of genes and the subcellular localization of gene products, which hinders the functional analyses of PI3K, PLC, and actin polymerization [37]. Nevertheless, our findings provide novel information about the regulation of tip growth in algae.

Our observations of generation and tip growth of branch initials in single-celled conchocelis from *P. yezoensis* indicate that PI3K and PLC activities are involved in both processes (Figure 1, Figure 2, Figures S1 and S2). Thus, we infer that phosphoinositide turnover activates signal transduction pathways at the branching point and apex of the stem cells and that this is necessary for the establishment and tip growth of branches in differentiated conchocelis cells. The cytoskeleton inhibitor CCB inhibited the growth of branches but not their initiation (Figure 3), whereas LAT-B inhibited both the initiation and growth of branches (Figure 4 and Figure S3). In fact, tip growth in single-celled conchocelis can be divided into four sequential events: generation of the branch initial on a differentiated conchocelis cell, elongation of the branch initial, initial cell division to establish the apical stem cell, and ongoing branch elongation based on division of the tip stem cells. Since it is well known that CCB and LAT-B inhibit F-actin-based G-actin polymerization and access of G-actin to F-actin, respectively, we speculate that the generation of the branch initial involves relocalizing pre-existing F-actin at the branch position in differentiated non-dividing conchocelis cells and does not require additional actin polymerization. In contrast, establishment of the apical stem cell by the first cell division in the new branch and the tip growth of the stem cell require new production and/or further accumulation of F-actin. Accordingly, we propose that the regulatory mechanisms for the establishment of the branch initial and tip growth of branches differ from those of hyphae-type tip growth, although F-actin, PI3K, and PLC are involved in both processes.

Despite the different effects of CCB and LAT-B on tip growth, the treatment of single-celled conchocelis with either inhibitor resulted in abnormal growth of branches and a wavy shape (Figure 3D and Figure 4D). A similar effect was observed after the treatment of conchocelis cells with PI3K and PLC inhibitors, although the effect was very weak, probably because of the short treatment time (see Figure 2C). Thus, we conclude that the determination of the growth direction is also important for normal tip growth; this and the required elongation and division of stem cells in the branch initials are all regulated by both phosphoinositide turnover and actin polymerization.

We previously demonstrated the involvement of phospholipase D (PLD) in the directional migration of asexual spores from *P. yezoensis*, for which asymmetrical F-actin distribution is indispensable [21]. It is obvious that PLD contributes to the maintenance of polarity, whereas PI3K-PLC cascade is necessary to establish polarity in spores [21]. Thus, although the present study did not mention a requirement for asymmetric F-actin distribution in tip growth, it is possible that PLD may be involved in branch elongation by maintaining the direction of cell division, rather than by contributing to the formation of the branch initial or the establishment of the apical stem cell during tip growth. Thus, in the near future, experiments addressing these possibilities will be necessary to confirm whether PLD functions in tip growth to increase knowledge about the critical involvement and functional diversity of phosphoinositide signaling cascades in tip growth of *P. yezoensis* conchocelis.

In conclusion, our study demonstrated that the activities of PI3K and PLC and the polymerization of actin are critically involved in tip growth in conchocelis cells of *P. yezoensis*. In addition, we have already demonstrated the critical involvement of auxin in the tip growth of these cells [18]. For auxin in *P. yezoensis*, we have demonstrated the presence of it in conchocelis cells [38] and the functional involvement of this plant hormone in tip growth in both conchocelis and conchosporangium [18], which is consistent with the involvement of auxin in the tip growth of the auxin-producing brown alga *Ectocarpus siliculosus* [3,39]. In terrestrial plants, auxin signal transduction plays important roles in tip growth [12,40,41,42,43,44,45,46,47]. In addition, it is well known that the auxin receptor transport inhibitor response 1 (TIR1) and transcription factors like *Auxin/Indole-3-Acetic Acid* (Aux/IAA) and auxin response factor (ARF) are the main components of auxin signal transduction [40,48,49]. However, *P. yezoensis* has no orthologs of genes encoding TIR1, Aux/IAA, and ARF, key factors in auxin signaling in terrestrial plants [38]. This suggests that red algae may have a novel pathway for auxin signal transduction. Thus, the identification of factors involved in auxin sensing and signal transduction is an important issue for elucidating the regulatory mechanisms of tip growth in *P. yezoensis*. Moreover, the relationships among auxin, actin polymerization, and phosphoinositide turnover in the regulation of tip growth are unknown in *P. yezoensis* at present. Thus, it will be valuable to determine whether the acceleration of actin polymerization and activation of PI3K and PLC are auxin dependent. To this end, developing methods for F-actin visualization in living cells and reverse-genetic techniques for analyzing gene functions in *P. yezoensis* is essential. These efforts could provide novel insights into the mechanisms regulating the generation of branch initials, establishment of stem cell, and tip growth of branches in conchocelis cells of *P. yezoensis*.

## 4. Materials and Methods

### 4.1. Algal Materials and Culture Conditions

The filamentous conchocelis of *P. yezoensis* (strain U-51) was maintained in sterilized artificial seawater, as in Li et al. [50], with weekly changes of seawater. Briefly, the culture conditions were 60 μmol photons m^−2^ s^−1^ light with a long-day photoperiod (14 h light/10 h dark) at 15 °C, with aeration with sterilized air filtered through a 0.22 μm filter (Whatman, Maidstone, UK).

### 4.2. Preparation of Single-Celled Conchocelis

As described by Taya et al. [18], aggregates of filamentous conchocelis were chopped using a razor blade, and single conchocelis cells were collected by filtration of fragmented conchocelis through a 10 µm nylon mesh to remove large pieces. The filtrate was subsequently incubated in 9 cm dishes (Asunoru dish, 90 mm (diameter) × 20 mm (height), As One Corporation, Osaka, Japan) containing 30 mL seawater at 15 °C for 10 min. Non-branched single conchocelis cells were identified using an Olympus IX73 light microscope (Olympus Corporation, Tokyo, Japan) equipped with an Olympus DP22 camera (Olympus Corporation, Tokyo, Japan), picked up using a micropipette, and transferred to 96-well plates (each cell/well containing 200 µL artificial seawater).

### 4.3. Treatment of Single-Celled Conchocelis with Inhibitors of PI3K and PLC

The cells were incubated in 200 µL artificial seawater containing 5 or 10 µM LY294002 (Cayman Chemical, Ann Arbor, MI, USA) or LY303511 (Bio-Techne, Minneapolis, MN, USA), which are active and inactive versions of PI3K-specific inhibitors, respectively, or containing 5 or 10 µM U73122 (Cayman Chemical, Ann Arbor, MI, USA) or U73433 (Cayman Chemical, Ann Arbor, MI, USA), which are active and inactive versions of PLC-specific inhibitors, respectively. Single-celled conchocelis was cultured statically in wells under the conditions described above, except for aeration. Control treatments included 1% DMSO (FUJIFILM Wako Pure Chemical Corporation, Osaka, Japan) without chemical inhibitors. The effects of the chemical inhibitors were evaluated after incubation for 3 days at 15 °C by calculating the branching rate (number of cells producing a branch as a percentage of the total number of cells observed) and measuring the length of the branches, both of which were performed by direct observation or by photographing the cells using an Olympus IX73 light microscope equipped with an Olympus DP22 camera.

### 4.4. Treatment of Single-Celled Conchocelis with Inhibitors of Actin Polymerization

The cells were incubated in 200 µL artificial seawater containing 10, 20, or 30 µM cytochalasin B (CCB; FUJIFILM Wako Pure Chemical Corporation, Osaka, Japan) or 5, 10, or 20 µM latrunculin B (LAT-B; AdipoGen Life Sciences, Basel, Switzerland), under the same conditions used for the PI3K and PLC inhibitor experiments. Control treatments included 1% DMSO (FUJIFILM Wako Pure Chemical Corporation, Osaka, Japan) without chemical inhibitors. After incubation for 7 days with CCB or LAT-B at 15 °C, the effects of these actin polymerization inhibitors were evaluated as above.

### 4.5. Statistical Analysis

Mean values ± SD were calculated from the triplicate experiments. Statistically significant differences in the interactions between the duration of incubation with the various chemicals and the regeneration rate of the branch initials or the length of branches were determined by one-way ANOVA with the Tukey−Kramer test (*p* < 0.05). Significant differences for each set of treatments were determined using a cutoff value of *p* < 0.05.

## Data Availability

Data are contained within this article or Supplementary Materials.

## Supplementary Materials

The following supporting information can be downloaded at: https://www.mdpi.com/article/10.3390/plants14142194/s1. Figure S1: Effects of LY294002 on tip growth. Figure S2: Effects of U793122 on tip growth. Figure S3: Recovery from the effects of latrunculin B (LAT-B) on tip growth after removing the inhibitor.
