# Supplementary material for: Phosphoinositide Signaling and Actin Polymerization Are Critical for Tip Growth in the Marine Red Alga Pyropia yezoensis
Source: Plants (Basel). 2025 Jul 15;14(14):2194. doi: 10.3390/plants14142194 (PMC12299721; doi:10.3390/plants14142194)
Supplement: Supplementary file 1 [file plants-14-02194-s001.zip › Figure S1.pdf]

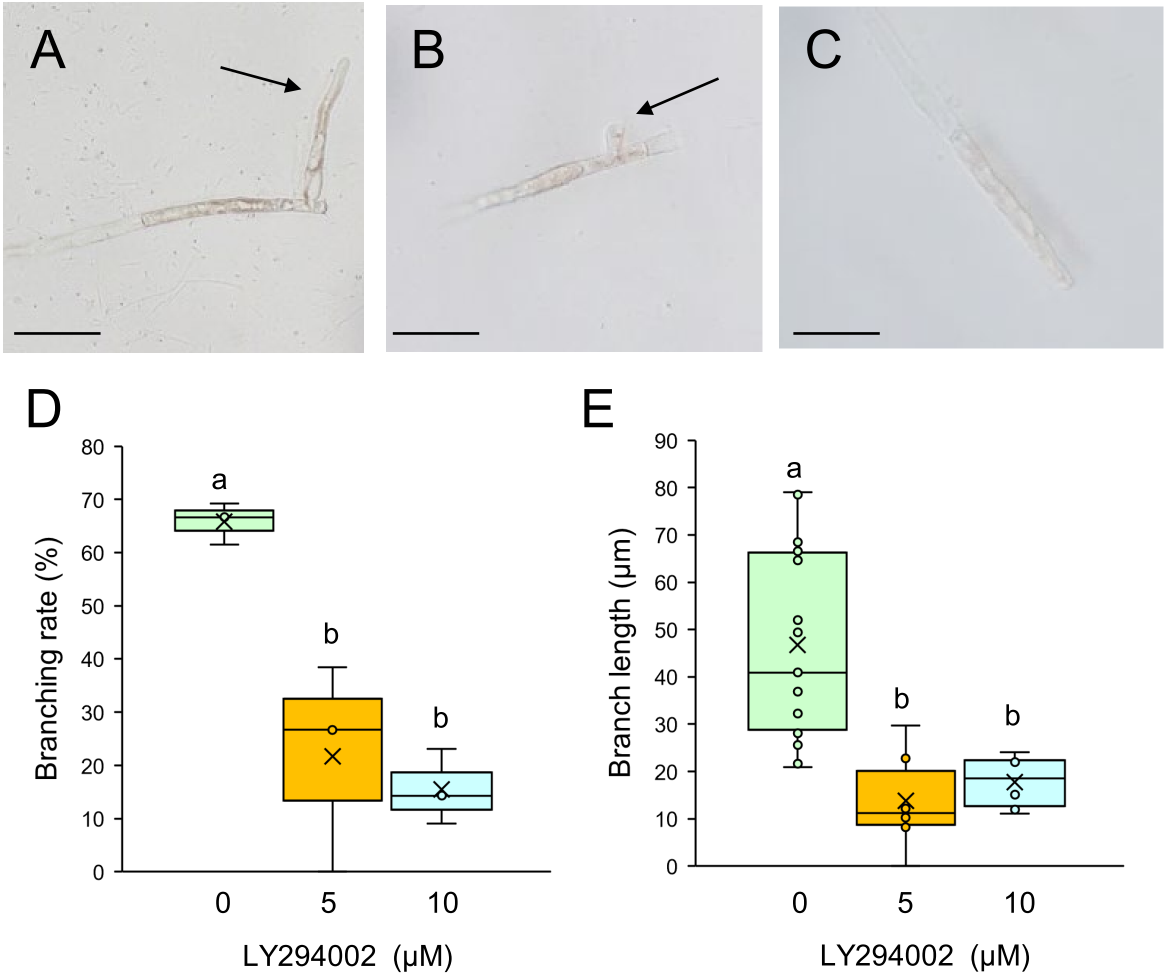

Figure S1. Effects of LY294002 on tip growth. (A–C) Photographs of single conchocelis cells treated with 0.5% DMSO (A) or 5 or 10 μM LY294002 (B, C) for 3 days. Arrows indicate newly generated branches. Bars: 25 μm. (D, E) Branching rate (D) and branch length (E) following treatment of single conchocelis cells with 0.5% DMSO (control) or 5 or 10 μM LY294002 for 3 days. Center line, median line; box limits, interquartile range with upper and lower quartiles; points, data; whiskers, range with maximum and minimum values; crosses, mean value. Lowercase letters denote significant differences in branching rate (D) and branch length (E) based on three independent experiments (n = 3) as determined by the Tukey-Kramer test (p < 0.05) for each set of treatments.
