# Supplementary material for: Phosphoinositide Signaling and Actin Polymerization Are Critical for Tip Growth in the Marine Red Alga Pyropia yezoensis
Source: Plants (Basel). 2025 Jul 15;14(14):2194. doi: 10.3390/plants14142194 (PMC12299721; doi:10.3390/plants14142194)
Supplement: Supplementary file 1 [file plants-14-02194-s001.zip › Figure S3.pdf]

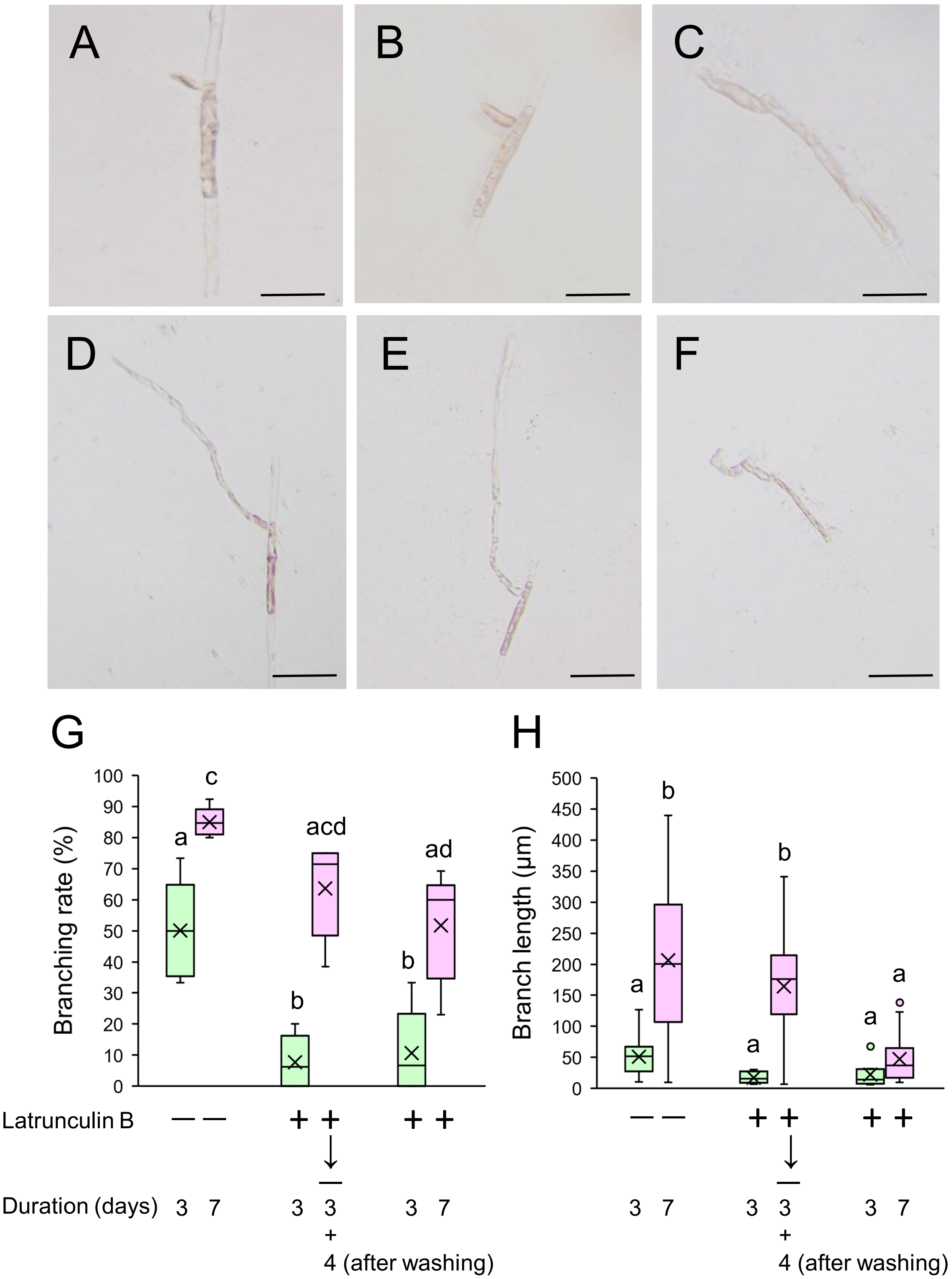
A
B
C
D
E
F
G
H
Branching rate (%)
100
90
80
70
60
50
40
30
20
10
0
a
c
acd
ad
b
b
Branch length (µm)
500
450
400
350
300
250
200
150
100
50
0
a
b
a
b
a
a
a
Latrunculin B
— —
+ +
+ +
Duration (days)
3 7
3 3 + 4 (after washing)
3 7

Figure S3. Recovery from the effects of latrunculin B (LAT-B) on tip growth after removing the inhibitor. (A–F) Photographs of single-celled conchocelis treated with 0.5% DMSO for 3 days (A), 15 μM LAT-B for 3 days (B, C), 0.5% DMSO for 7 days (D), 15 μM LAT-B for 3 days followed by washing to remove the inhibitor and incubation for another 4 days (E), and 15 μM LAT-B for 7 days (F). Bars: 25 μm (A–C) and 50 μm (D–F). (G, H) Branching rate (G) and branch length (H) following treatment of single conchocelis cells with 0.5% DMSO for 3 or 7 days as controls (2 bars at left); 15 μM LAT-B for 3 days followed by washing to remove the inhibitor and incubation with 0.5% DMSO for 4 days (2 bars at center; indicated by + → —); and 15 μM LAT-B for 3 or 7 days (2 bars at right). Center line, median line; box limits, interquartile range with upper and lower quartiles; points, data; whiskers, range with maximum and minimum values; crosses, mean value. Lowercase letters denote significant differences in branching rate (G) and branch length (H) based on three independent experiments (n = 3) as determined by the Tukey-Kramer test ($p < 0.05$) for each set of treatments.
